# A qualitative study on harmonization of postgraduate medical education in Europe: negotiating flexibility is key

**DOI:** 10.1007/s40037-019-0523-4

**Published:** 2019-07-11

**Authors:** Jessica E. van der Aa, Fedde Scheele, Angelique J. Goverde, Pim W. Teunissen

**Affiliations:** 1 Department of Research and Education, OLVG Hospital, Amsterdam, The Netherlands; 2 Athena Institute, Faculty of Science, VU University, Amsterdam, The Netherlands; 3 European Board & College of Obstetrics and Gynaecology, Brussels, Belgium; 4 Department of Obstetrics and Gynecology, Amsterdam UMC, VU University Medical Centre, Amsterdam, The Netherlands; 5 Department of Reproductive Medicine and Gynaecology, University Medical Centre, Utrecht, The Netherlands; 6 School of Health Professions Education (SHE), Faculty of Health Medicine and Life Sciences, Maastricht University, Maastricht, The Netherlands

**Keywords:** Postgraduate medical education, Harmonization, Curriculum development

## Abstract

**Introduction:**

International harmonization of postgraduate medical education is gaining importance in the globalization of medical education. Harmonization is regarded as the establishment of common standards in education, while maintaining regional or local freedom to adapt training to contexts. During the development of a harmonized curriculum, tensions between standardization and contextualization may surface. To allow future harmonization projects to recognize these tensions and deal with them in a timely manner, this study aims to gain insight into tensions that may arise when developing a harmonized curriculum for postgraduate medical education in Obstetrics and Gynaecology in Europe.

**Methods:**

Focus groups were held with international curriculum developers to discuss challenges that resulted from tensions between standardization and contextualization when developing a harmonized European curriculum for postgraduate medical education in Obstetrics and Gynaecology. Data were analyzed through conventional content analysis, using the principles of template analysis.

**Results and Discussion:**

Tensions between standardization and contextualization in the development of a harmonized curriculum were apparent in two domains: 1) Varying ideas about what the harmonized curriculum means for the current curriculum and 2) Inconsistencies between educational principles and the reality of training. Additionally, we identified ways of dealing with these tensions, which were characterized as ‘negotiating flexibility’. Tensions between standardization and contextualization surfaced in the development phase of harmonizing a curriculum, partly because it was anticipated that there would be problems when implementing the curriculum.

## What this paper adds

Tensions between standardization and contextualization are experienced in medical education. They may surface in the development of a harmonized curriculum in particular, which may challenge the process since harmonization is aimed at providing standards while respecting local autonomy. This study is the first to gain insight into the tensions that may arise when developing a harmonized curriculum for postgraduate medical education in Europe, to allow future harmonization projects to recognize these tensions and deal with them in a timely manner.

## Introduction

Globalization of medical education has received growing attention [1–7]. While globalization efforts are being realized, concerns are raised about the feasibility of development and implementation of international standards in different contexts [2, 5, 6]. International harmonization of medical education is a form of globalization, since a harmonized curriculum can be developed for implementation in different contexts, such as different countries [7]. Several European medical specialty societies have started the process of international harmonization of postgraduate medical education [8–13] to improve the quality of education and care, and to enhance mobility of medical specialists throughout Europe [12, 14, 15]. Patrício and Harden explain that in a harmonization process common standards in education are established, while regional or local autonomy is maintained [16]. However, this may be challenging at an international level, especially if multiple healthcare systems and different cultures are involved [1, 17, 18].

In medical education in general, balancing standardization of education with allowances for contextual variation is increasingly a subject of debate [5, 19]. Bates et al. discussed how processes of standardization and existing contextual diversity intersect in different areas of medical education and how this may cause tensions between the two [20]. Potentially, these tensions may also arise when harmonizing postgraduate medical education. This deserves exploration because, as we have stated, globalization and harmonization efforts are currently being undertaken, and challenges are encountered along the way [5, 7, 21]. A better understanding of potential tensions in the process of harmonization and how they are negotiated is necessary to understand and address potential challenges in the process [22].

Before we can build on this debate, the terms ‘standardization’ and ‘contextualization’ require further explanation. For the purpose of harmonizing postgraduate medical education, standardization of training refers to the use of common learning outcomes, strategies for training, and systems of assessment, for instance in an outcome-based curriculum [9, 23]. In postgraduate medical education, it is essential to align standards of training with contexts of training. The context of postgraduate medical education is the workplace, which is shaped by patient care but also by social relations, by organization of healthcare and by clinical practice [24]. The workplace offers continuous variation in learning opportunities for trainees [25]. Workplace-based learning helps trainees to develop the required competencies for their profession through exposure and interaction in the workplace [19, 25]. Making optimal use of the workplace while aligning standards of training with workplace-based contexts is regarded as contextualization of postgraduate medical education.

As suggested, tensions between standardization and contextualization may surface in medical education, especially when harmonizing postgraduate medical education. An important phase in harmonizing postgraduate medical education is curriculum development. Ideally, in this phase, curriculum developers recognize challenges and deal with them to ensure feasibility and effective implementation of the curriculum. Therefore, we aimed to explore if and how tensions between standardization and contextualization surface during the development of a harmonized curriculum in postgraduate medical education to better understand the process of harmonization and the challenges that it may bring. We do not aim to find straightforward solutions, rather we are convinced that understanding the challenges in harmonization may offer starting points for consideration to improve feasibility and sustainability of harmonization of postgraduate medical education in the future. Thus, taking into account the increasing globalization of medical education, this research is valuable for future projects and the evolvement of globalization.

Recently, the European Board & College of Obstetrics and Gynaecology (EBCOG) took steps to harmonize postgraduate medical education in Obstetrics and Gynaecology (OBGYN) in Europe [12]. We took this project as an opportunity to investigate how tensions between standardization and contextualization surface in the development of a harmonized curriculum to consider the feasibility of the European OBGYN curriculum before it is implemented.

The research questions were: 1) Do curriculum developers recognize and/or deal with tensions between standardization and contextualization in the development of a harmonized curriculum in postgraduate medical education, and if so 2) Where do such tensions surface?’

## Methods

We performed a conventional content analysis study with focus groups to gain an understanding of curriculum developers’ experiences of tensions between standardization and contextualization during the development of the European OBGYN curriculum [26]. In focus groups with curriculum developers, we investigated their recognition of, and experience with, the tensions between standardization and contextualization that they encountered during the phase of curriculum development. We organized focus groups because they allow participants to build on each other’s perspectives through group interaction.

### Participants and setting

As explained, EBCOG has initiated the harmonization of postgraduate medical education in OBGYN in Europe [12]. Participants in this project were members of EBCOG, members of the European Network of Trainees in Obstetrics and Gynaecology (ENTOG), representatives of subspecialty societies in OBGYN, educationalists and representatives of stakeholders of postgraduate medical education in OBGYN (i.e. patients, midwives, nurses, and hospital board members) [12]. In total, the group of curriculum developers consisted of 38 people.

All 38 curriculum developers were identified as potential participants for this study and were contacted by email. They received information about the study and an invitation to participate. To include as many of the curriculum developers as possible, the focus groups were scheduled during international project meetings. The first focus group consisted of the advisory committee of the project, representing EBCOG members and members of subspecialty societies in OBGYN. The second focus group consisted of ENTOG members, representing trainees in OBGYN from different European countries. Trainees were deliberately invited as a separate group to prevent hierarchical relations affecting group dynamics. The third focus group consisted of the project’s working group, including gynaecologists, trainees, educationalists and representatives of stakeholder groups. During each focus group session, two moderators facilitated the discussion (FS & JEA or AJG & JEA). All focus groups were held in English, which is the second language of most participants.

The focus group moderators encouraged participants to share any challenges they had experienced during curriculum development. A lot of room was offered for reflection and elaboration. The topic guide of the focus groups was limited to the challenges experienced, although this was slightly structured by suggesting that challenges may be experienced at three levels (macro, meso and micro). The moderators made no direct references to the concept of tensions between standardization and contextualization, since this was considered too conceptual and too restrictive for collection of valuable data. The topic guide did not evolve throughout the study, although the discussions in the three focus groups were very divergent because of their free flowing nature.

Eventually, three focus groups were held, each with a duration of approximately one hour. In the first, second and third focus group there were seven, four and nine participants, respectively. The total of twenty participants represented twelve different European nationalities.

### Data analysis

Data were collected between November 2017 and February 2018. The focus groups were audio recorded and transcribed verbatim. Audio recordings and transcripts were stored in a secured environment. After analyzing the data, the recordings were destroyed. The main researcher (JEA) moderated all three focus groups and coded the transcripts with the help of MaxQDA2007. We performed conventional content analysis according to the steps of King’s template analysis to allow structured and hierarchal organization of the coding [27]. Through open coding, an initial codebook was developed by the main researcher (JEA). Coding concentrated on recognizing specifically mentioned challenges or struggles as experienced by the curriculum developers in relation to the development of a curriculum that was intended to be suitable for many different training contexts. Subsequent template analysis resulted in identification of domains of tension [27]. JEA discussed the domains with the three other researchers through an iterative process until consensus was reached about the nature and interpretation of the domains.

### Ethical considerations and reflexivity

Participants were contacted in person and informed about the purpose of the study, the role of the participant in the study and the reason for selecting the participant. The voluntary nature of participation was stressed. The researchers ensured that participants were aware that if they decided to withdraw from participation after the focus groups had taken place, their input could not be eliminated from analysis. Also, the researchers stressed the confidential nature of the information that was shared within the focus groups. Consent for participation in the study was obtained orally and in writing and there was an opportunity for questions from participants and further explanation by the researchers. There were no personal advantages nor possible risks in participating in this study. The study was approved by the Ethical Review Board of the Netherlands Association for Medical Education in 2016 (NVMO-ERB file number 795). Only the main researcher (JEA) had access to the original data, and all research material was coded to guarantee anonymity.

Three of the researchers (JEA, FS, AJG) were also involved in developing the European OBGYN curriculum. Although they did not participate in the focus groups but moderated them, the study may have been influenced by their positions, beliefs and values regarding harmonization of postgraduate medical education in OBGYN. For instance, the main researcher (JEA) initially believed that harmonizing postgraduate medical education would improve the quality of training in all European countries. However, her involvement in exploring the process as a researcher resulted in new insights that have considerably changed her initial stance. The majority of the participants also shared a positive perspective on harmonization, which was underlined by their involvement in the development of the European OBGYN curriculum. Variation in their geographical and professional backgrounds ensured that the issues under study were addressed from different contexts.

## Results

Analysis of the data revealed that the developers of the European OBGYN curriculum recognized tensions between standardization and contextualization during the development of the harmonized curriculum. We identified two domains in which these tensions were apparent. Additionally, we identified ways to deal with the tensions that emerged from our data. Whenever we refer to the ‘harmonized curriculum’, we mean the European OBGYN curriculum that was developed for the purpose of harmonization of postgraduate medical education in OBGYN in Europe. Whenever we refer to the ‘current curriculum’ we mean the OBGYN curriculum of a country or region with which the participants were familiar.

### Domain 1: Varying ideas about what the harmonized curriculum means for the current curriculum

Throughout the process of curriculum development, the harmonized curriculum gradually began to take shape. In this process, the participants automatically compared the harmonized curriculum with their current curriculum. Participants increasingly empathized with the way people from their country or region might ‘feel’ about the harmonized curriculum, which was based on participants’ understanding of the current curriculum. This ‘feeling’ was identified as a general feeling about the entire harmonized curriculum, rather than about specific elements of the harmonized curriculum. This gave participants an idea of how their current curriculum should change, in order for it to comply with the harmonized curriculum. While participants discussed these ideas with each other, they discovered that there were differences between countries. To illustrate this, we provide three examples of different ideas about the harmonized curriculum and we explain which strategies for change are implied in these ideas.

For instance, one participant thought that her colleagues would mainly compare their current curriculum with the harmonized curriculum to confirm the quality of their current curriculum. In her view, medical educators in her country only need confirmation that their curriculum is already of high quality and therefore do not need the harmonized curriculum to show how to improve their current training. This was supported by the following quote:

*I think in [country] there will not be a huge change and they won’t be open for a huge change because they have an elaborate curriculum and they will stay with it. Because there is a lot of work behind [the current curriculum] and it is established.*

Another participant explained that she felt that her country considered the harmonized curriculum to be a benchmark to show which elements of the current curriculum could be improved. Therefore, her idea for evolvement of their current curriculum was that selected elements of training might be improved, for which the harmonized curriculum would be a good reference. For yet another country, a participant explained that she felt that the harmonized curriculum is so far from her country’s current curriculum that her idea of change towards the current curriculum is thorough reform and extensive improvement.

These three examples reveal that tension is experienced between standardization and contextualization, since participants collectively struggled with the realization that there were different ideas about what the harmonized curriculum means for the current curriculum.

### Domain 2: Inconsistencies between educational principles and the reality of training

Throughout curriculum development, specific educational principles were selected as the foundation for the harmonized curriculum. Examples of educational principles that the harmonized curriculum relies on are an outcome-based curriculum, a soft-skills framework, a focus on simulation and teamwork training and assessment through entrustment to ensure gradual independence.

Participants explained that in their experience, some principles might not be consistent with the reality of training in their constituent workplaces and some principles might even have an adverse effect if they were implemented in specific countries. For instance, participants described that one of the educational principles of the harmonized curriculum is that a trainee should gradually gain independence in practice, and that this should be officially documented in a portfolio using entrustment decisions. One trainee feared that by formalizing this process of gaining gradual independence, some supervisors might be more hesitant to leave trainees to practice independently, rather than to stimulate it, because of the formal process:

*If the professor or specialist see I can do it, he will leave me do it. But if he has to sign I’m confident to do it, then maybe he will start making problems about it. Because he will put the signature there and then feels responsible for it. So it takes longer to reach the competences that already have [the competences] informal*.

This type of tension between standardization and contextualization was recognized in more cases. As a second example, two participants pointed out that the harmonized curriculum describes a broad standard of general competencies in the curriculum content, while at the same time the current workforce of gynaecologists in Europe seems to be differentiating and subspecializing more and more. According to the participants, this tendency to subspecialize would lead to a workplace that offers mainly learning opportunities that are more subspecialty-specific, which may compete with learning opportunities for the development of more general competencies. This view was supported by the following quote:

*Staff members, (…) they are subspecialized more and more nowadays to teach their trainees, again, about advanced things in perinatal medicine or reproductive medicine, urogynaecology. And obviously that’s going to be rectified now in this new curriculum. But it needs again a change of perception and that may in itself be, also be a challenge to change that*.

### Negotiating flexibility

In addition to the two domains of tensions, we identified in our data that participants already tried to deal with the tensions in the curriculum development phase. Participants’ understanding of the existence of tensions seemed to initiate considerations of how to deal with them. Participants suggested that curriculum developers should engage in conversations with national postgraduate medical education bodies as well as local sites to explore the different needs of the different contexts. One participant explained:

*But still we do visits in many, many countries in Europe. And that also may contribute to getting more information as to how new curriculums will be received and how they think they will able to fit it into let’s say a local organization*.

Participants experienced that consideration of differences subsequently led to their understanding that flexibility of the harmonized curriculum is needed to allow the standards to align with different contexts. Therefore, the harmonized curriculum should be developed further, based on more understanding of specific needs for flexibility. Participants described the harmonized curriculum as a ‘document in progress’ for which flexibility should be negotiated. This is supported by the following quotes:

*I think that’s important to talk about to each other and discuss about it. Are we doing it as we thought we are doing it?*

*And within the tailoring process there might be a stepwise change of course*.

For instance, one of the participants remarked that a ‘pick-and-choose’ strategy for specific elements of the harmonized curriculum may undermine the desired effects of quality improvement of postgraduate medical education throughout Europe. This is a consideration of flexibility that requires negotiation.

*And then the other thing is if, for example, a country says, we can’t actually deliver that part of the curriculum, does that matter?*

Overall, participants agreed that the harmonized curriculum should be regarded as a strong recommendation for OBGYN training, while it should still allow for flexibility to align it with different contexts. This shows that the curriculum developers experienced tensions between standardization and contextualization when developing the harmonized curriculum, but also searched for ways to deal with them, and felt that negotiating flexibility of recommendations deals with these tensions. The following quote stresses how participants perceived implementation and further development of the harmonized curriculum.

*If you also accept that things take time. And maybe it will take five to ten years to reach [it]. And everyone will have their own from now on until ten years. And you have to accept that. The squares getting rounder and rounder you know? You have to accept that, because change, management of change is really hard*.

## Discussion

The aim of this study was to gain insight into tensions that may arise in the development of a harmonized curriculum for postgraduate medical education in Europe. Our findings show that curriculum developers recognized tensions between standardization and contextualization when developing a harmonized curriculum. Tensions surfaced due to varying ideas about what the harmonized curriculum means for the current curriculum, and due to inconsistencies between educational principles and the reality of training. Also, curriculum developers shared how these tensions may be dealt with.

Reflecting further on the first domain in which tensions surfaced, we identified a challenge that requires some further exploration. The objective of the development of the harmonized curriculum is to improve training throughout Europe, although the tensions in the first domain show that different countries have different ideas about what the harmonized curriculum means for the current curriculum. This raises the question whether the intention to improve training across Europe can be fulfilled through harmonization at all. We wonder whether harmonization could unintentionally lead to an increase of inequality in postgraduate medical education if some countries evolve towards the harmonized curriculum easily, whereas other countries need more time and effort to change. The countries that need to invest more time and effort to adopt the harmonized curriculum need to initiate their development while being aware of the size of the effort. This awareness may affect a country’s perception of harmonization in a negative way and potentially lead to undermining its efforts even further. For countries that do not need to invest so much time and effort to change towards the harmonized curriculum, harmonization may seem a more achievable aim. In a harmonization process it is important to be aware of these different effects on countries. Negotiating room for flexibility within a harmonized curriculum allows for better alignment of the harmonized curriculum to different contexts and makes adaptation of current curricula more feasible.

This study originated from discussions around tensions between standardization and contextualization in medical education in general. In their paper, Bates et al. recommended to embrace both standardization and contextualization as valuable concepts, accepting the tensions they cause, and we should aim to deliver medical education that is both globally responsible and locally engaged [20]. Relating our research to this literature, we believe that our findings resonate with Bates’ statement on embracing both concepts. Our study shows that recognizing tensions is part of the process of harmonization. The need for negotiating room for flexibility of the standards, without necessarily eliminating tensions, to fit different contexts, has become evident.

Literature regarding change processes in postgraduate medical education curricula describes how expectations of a novel curriculum are not always met during implementation in the context [28–32]. What we have learned in our study is that with regard to the harmonization of postgraduate medical education, tensions between standardization and contextualization can be recognized in the curriculum development phase, thus already prior to implementation. However, in reforming medical education curricula the phases of curriculum development and implementation are not linear. Often the two phases are intertwined and interact with one another [28, 31]. Curriculum developers in this study anticipated potential implementation challenges and this influenced the curriculum development. There is, however, a risk with this approach, since some of the early anticipated problems may seem less necessary to be resolved at a later stage. It is uncertain to what extent curriculum developers should anticipate potential challenges for implementation. This requires careful consideration.

The findings of this study may benefit the harmonization of postgraduate medical education in the future. We propose a strategy to allow flexibility of a harmonized curriculum. That is, to consider if a harmonized curriculum could be developed at two levels. For instance, a harmonized curriculum can describe educational outcomes and strategies for all the countries involved. In addition, all countries may describe how they intend to interpret and ensure implementation of the harmonized curriculum in the context of training. For a harmonized curriculum to serve different contexts, local interpretation may be necessary. This two-level approach of curriculum development may answer the need for local flexibility, while still allowing for harmonization of postgraduate medical education.

### Strengths, limitations and future research

The strength of this study is that it provides a novel insight into tensions between standardization and contextualization in the development of a harmonized curriculum for postgraduate medical education in Europe. As explained, three of the researchers as well as most participants in this research share a positive conception around harmonization. This may have affected the outcomes of the study. Future research could be aimed at further exploring more deeply seated tensions, by including participants with potentially more diverging conceptions around harmonization.

The findings of this study open up possibilities for further research. More empirical evidence is required on the value of the suggested negotiation of flexibility through a two-level approach of curriculum development. Dealing with tensions between standardization and contextualization when harmonizing postgraduate medical education is an investment whose benefits are not clear yet. As previously described, harmonization of postgraduate medical education aims to enhance the international exchange of best practices in healthcare as well as the mobility of medical specialists [12]. Further qualitative research is needed to provide insight into whether these aims are met and under which conditions.

## Conclusions

Curriculum developers recognize tensions between standardization and contextualization in the development of a harmonized curriculum. Tensions may surface due to varying ideas about what the harmonized curriculum means for the current curriculum, and due to inconsistencies between educational principles and the reality of training. Negotiating room for flexibility in anticipation of national implementation challenges seems to be the main strategy to deal with perceived tensions in the harmonization of postgraduate medical education.
